# Supplementary material for: The mitochondrial phylogeny of land plants shows support for Setaphyta under composition-heterogeneous substitution models
Source: PeerJ. 2020 Apr 28;8:e8995. doi: 10.7717/peerj.8995 (PMC7194085; doi:10.7717/peerj.8995)
Supplement: Supplemental Information 4 [file peerj-08-8995-s004.pdf]

Supporting Information - The mitochondrial phylogeny of land plants shows support for Setaphyta under composition-heterogeneous substitution models

Filipe de Sousa, Peter Civáň, João Brazão, Peter G. Foster and Cymon J. Cox

Results

Analyses of the 36 gene, 26 taxa dataset

GENE LIST

Gene	length(bp)	n taxa	comp. vectors (nt)	p-value CV1 (nt)	p-value CV-n (nt)	length(aa)	comp. vectors (aa)	p-value CV1 (aa)	p-value CV-n (aa)
atp1	1422	26	CV2	0.0000	0.825	474	CV2	0.0000	0.1401
atp4	534	23	CV2	0.0000	0.163	178	CV2	0.0005	0.1941
atp6	756	26	CV2	0.0000	0.202	252	CV2	0.0000	0.4748
atp8	456	24	CV2	0.0000	0.448	152	CV2	0.0009	0.1995
atp9	222	25	CV2	0.0000	0.635	74	CV2	0.0356	0.4698
ccmB	624	18	CV2	0.0000	0.2775	208	CV2	0.0000	0.0931
ccmC	663	18	CV2	0.0000	0.5925	221	CV2	0.0000	0.4584
cob	1140	26	CV2	0.0000	0.7125	380	CV2	0.0000	0.4272
cox1	1566	26	CV2	0.0000	0.3785	522	CV2	0.0000	0.3619
cox2	756	26	CV2	0.0000	0.4675	252	CV1	0.2837	0.2837
cox3	795	26	CV2	0.0000	0.5755	265	CV2	0.0000	0.3218
nad1	945	26	CV2	0.0000	0.5225	315	CV2	0.0005	0.6257
nad2	1467	26	CV2	0.0000	0.713	489	CV2	0.0000	0.1817
nad3	354	26	CV2	0.0000	0.5045	118	CV2	0.0015	0.3678
nad4	1482	26	CV2	0.0000	0.433	494	CV2	0.0000	0.2371
nad4L	300	26	CV2	0.0000	0.422	100	CV2	0.0188	0.2055
nad5	2001	26	CV2	0.0000	0.546	667	CV4	0.0000	0.0500
nad6	597	26	CV2	0.0000	0.2345	199	CV2	0.0000	0.2035
nad7	1179	22	CV2	0.0000	0.499	393	CV2	0.0000	0.1480
nad9	555	26	CV2	0.0000	0.476	185	CV1	0.1074	0.1074
rpl16	405	22	CV2	0.0000	0.5635	135	CV1	0.2069	0.2069
rpl2	1053	22	CV2	0.0000	0.967	351	CV2	0.0009	0.7238
rpl5	558	23	CV2	0.0000	0.3925	186	CV2	0.0129	0.3109
rpl6	303	18	CV2	0.0040	0.781	101	CV1	0.4154	0.4154
rps1	492	22	CV2	0.0000	0.3195	164	CV2	0.0292	0.3500
rps10	276	15	CV2	0.0005	0.4015	92	CV1	0.4842	0.4842
rps11	333	23	CV2	0.0015	0.2225	111	CV1	0.4673	0.4673
rps12	375	25	CV2	0.0000	0.9825	125	CV1	0.2609	0.2609
rps13	336	23	CV2	0.0000	0.455	112	CV1	0.1055	0.1055
rps14	297	23	CV2	0.0000	0.429	99	CV1	0.4094	0.4094
rps19	252	21	CV2	0.0000	0.2035	84	CV1	0.7941	0.7941
rps2	630	22	CV2	0.0000	0.5185	210	CV1	0.3005	0.3005
rps3	507	24	CV2	0.0000	0.63	169	CV2	0.0089	0.1539
rps4	588	26	CV2	0.0000	0.3575	196	CV3	0.0000	0.0629
rps7	387	23	CV2	0.0000	0.792	129	CV1	0.3946	0.3946
sdh4	258	22	CV2	0.0000	0.1925	86	CV1	0.3837	0.3837

Table S.1 The list of 36 mitochondrial genes showing the sequence length in base pairs (bp) and amino acid (aa) and the number of taxa in each gene. The number of composition vectors that fits the data is shown for both nucleotide (nt) and amino acid (aa) alignments. The p-value of the X^2 distribution of composition homogeneity is presented for the homogeneous (CV1) analysis and for the best-fitting (CV-n) analysis, for both nucleotide and amino acid alignments.

Phylogenetic Trees

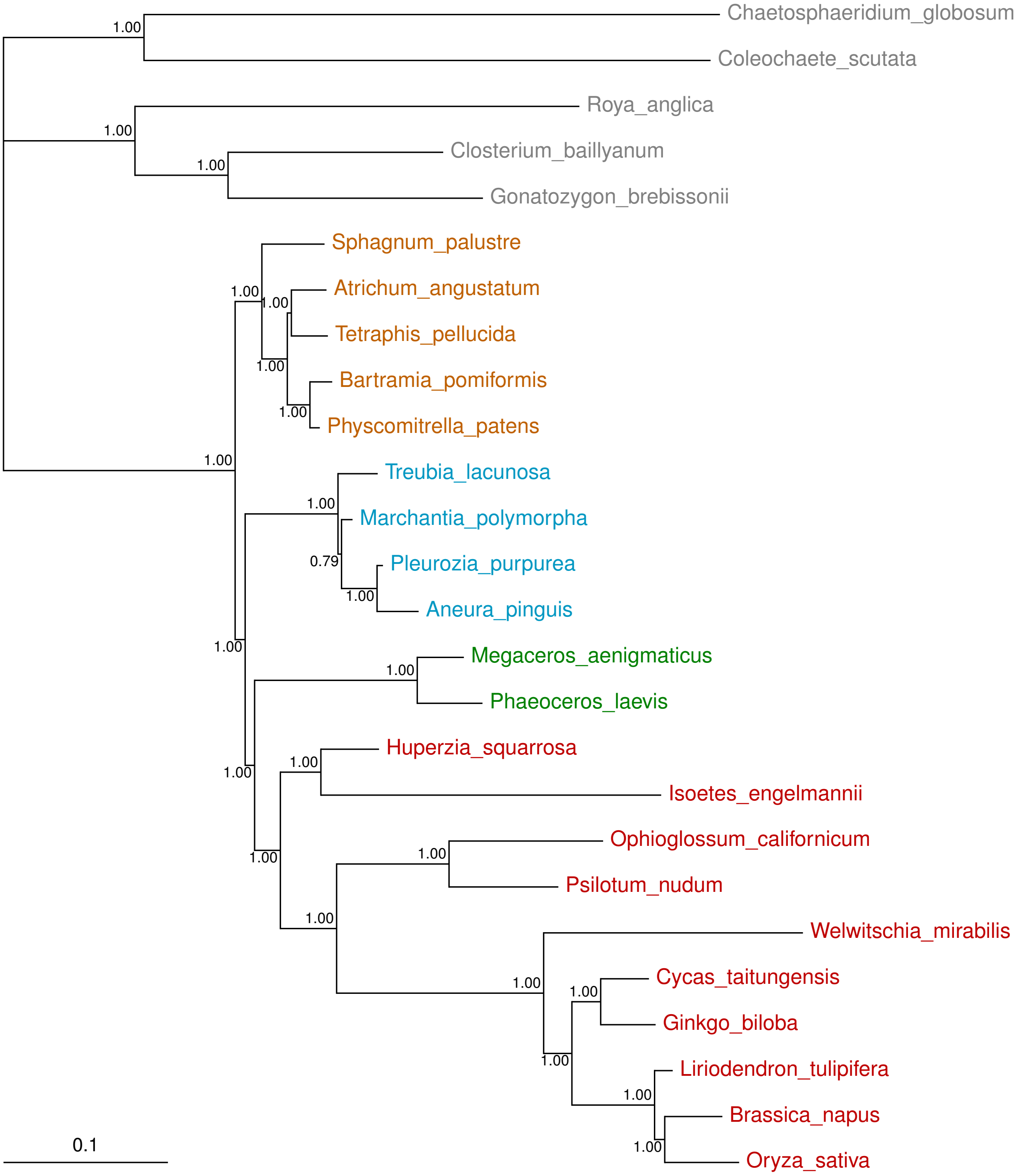

Figure S1 **36 genes/26 taxa** MCMC of the 36 gene nucleotide matrix using the model GTR+G with one composition vector (CV1). The tree-homogeneous composition model does not fit the expectation from posterior predictive simulations (p= 0.0). The tree shows full support for early-diverging mosses (PP= 0.99). Runs x2. 1M generations, burnin= 5000. $GTR + \Gamma_4 + F_{est}$ Marginal likelihood: $-\overline{L}_h$= 240782.2540. ASDOSF= 0.0132. Posterior predictive simulations of X^2: original statistic= 4649.2199, sample distribution= 8.5315 to 61.5624, p= 0.0000

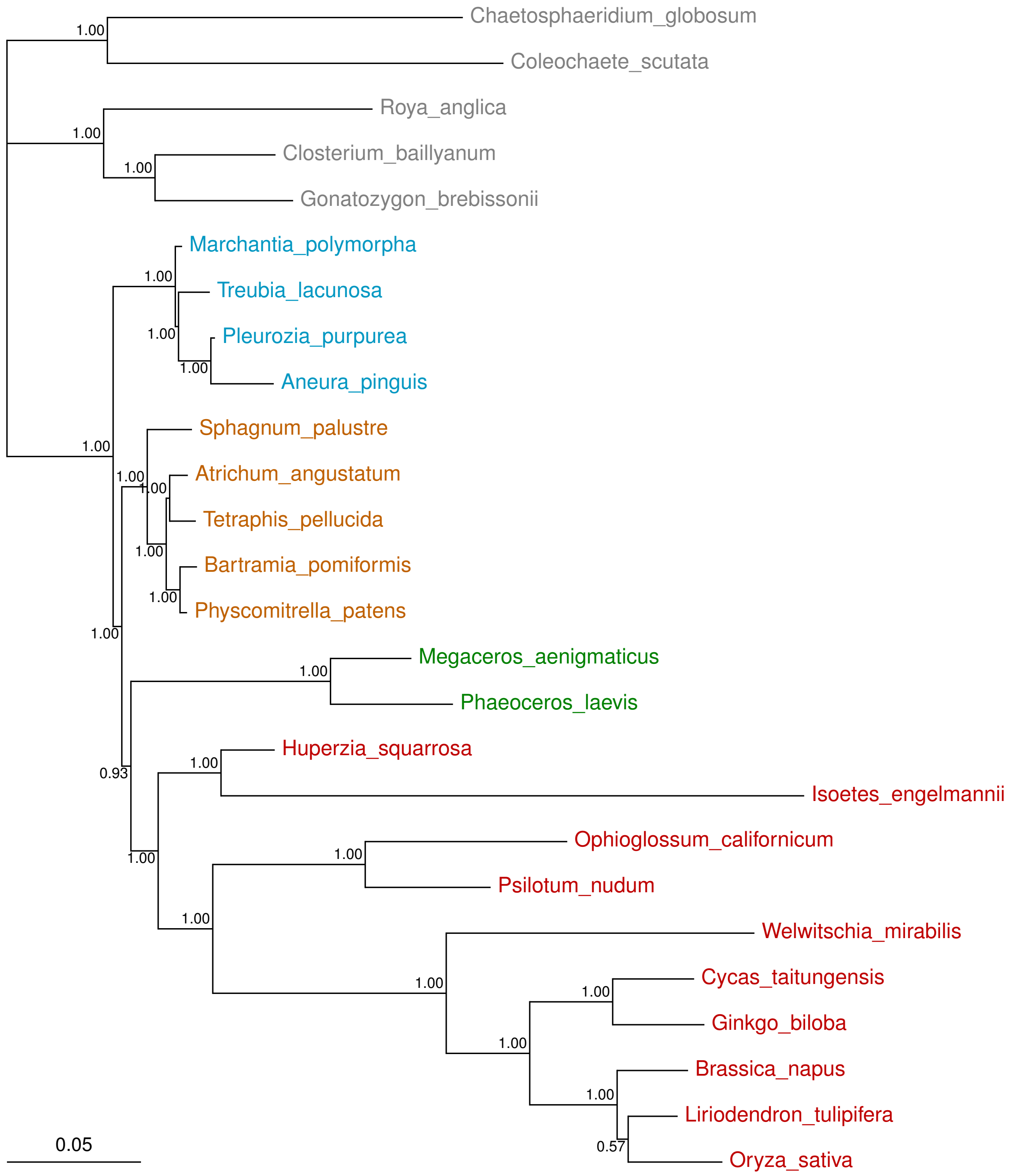

Figure S2 36 genes/26 taxa MCMC of the 36 gene degenerate nucleotide matrix using the model GTR+G with one composition vector (CV1). The tree-homogeneous composition model does not fit the expectation from posterior predictive simulations (p= 0.0). The tree shows early-diverging liverworts (PP= 1.0). Runs x2. 1M generations, burnin= 6000. $GTR + \Gamma_4 + F_{est}$ Marginal likelihood: $-\overline{L}_h$= 123024.6111. ASDOSF= 0.0115. Posterior predictive simulations of X^2: original statistic= 3690.7160, sample distribution= 5.5779 to 37.3095, p= 0.0000

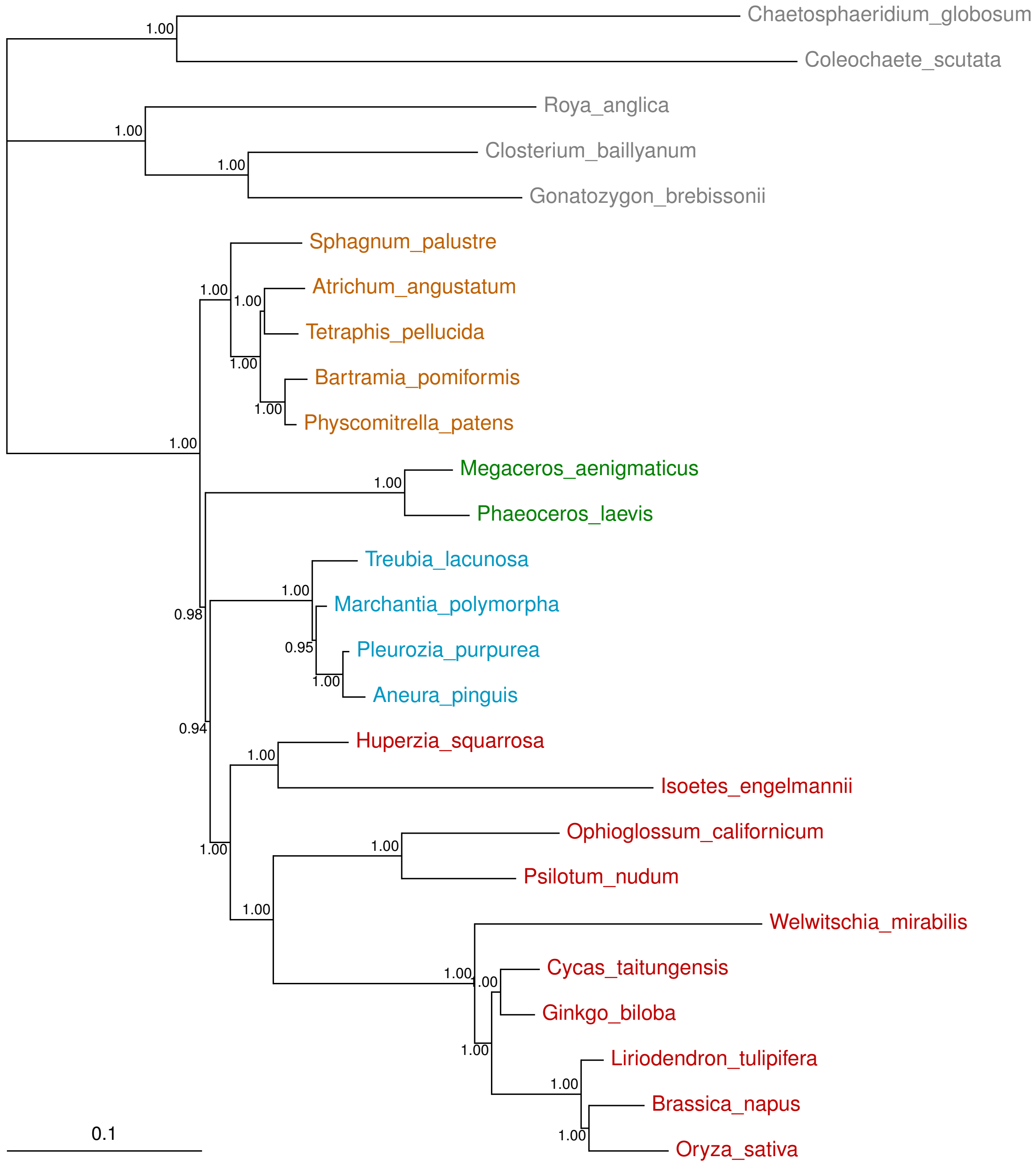

Figure S3 **36 genes/26 taxa** MCMC of the 36 gene nucleotide matrix using the model GTR+G and the NDCH2 model. The tree-heterogeneous composition model fits the expectation from posterior predictive simulations (p= 0.99). The consensus tree from analysis with the best marginal likelihood (r6) shows early-diverging mosses (PP= 0.98). Runs x2. 3M generations, burnin= 21000. $GTR + \Gamma_4 + F_{est}$ Marginal likelihood: $-\overline{L}_h$= 235706.7733; ASDOSF= 0.0287. Posterior predictive simulations of X^2: original statistic= 4649.2199, sample distribution= 4579.9114 to 6208.1942, p= 0.99

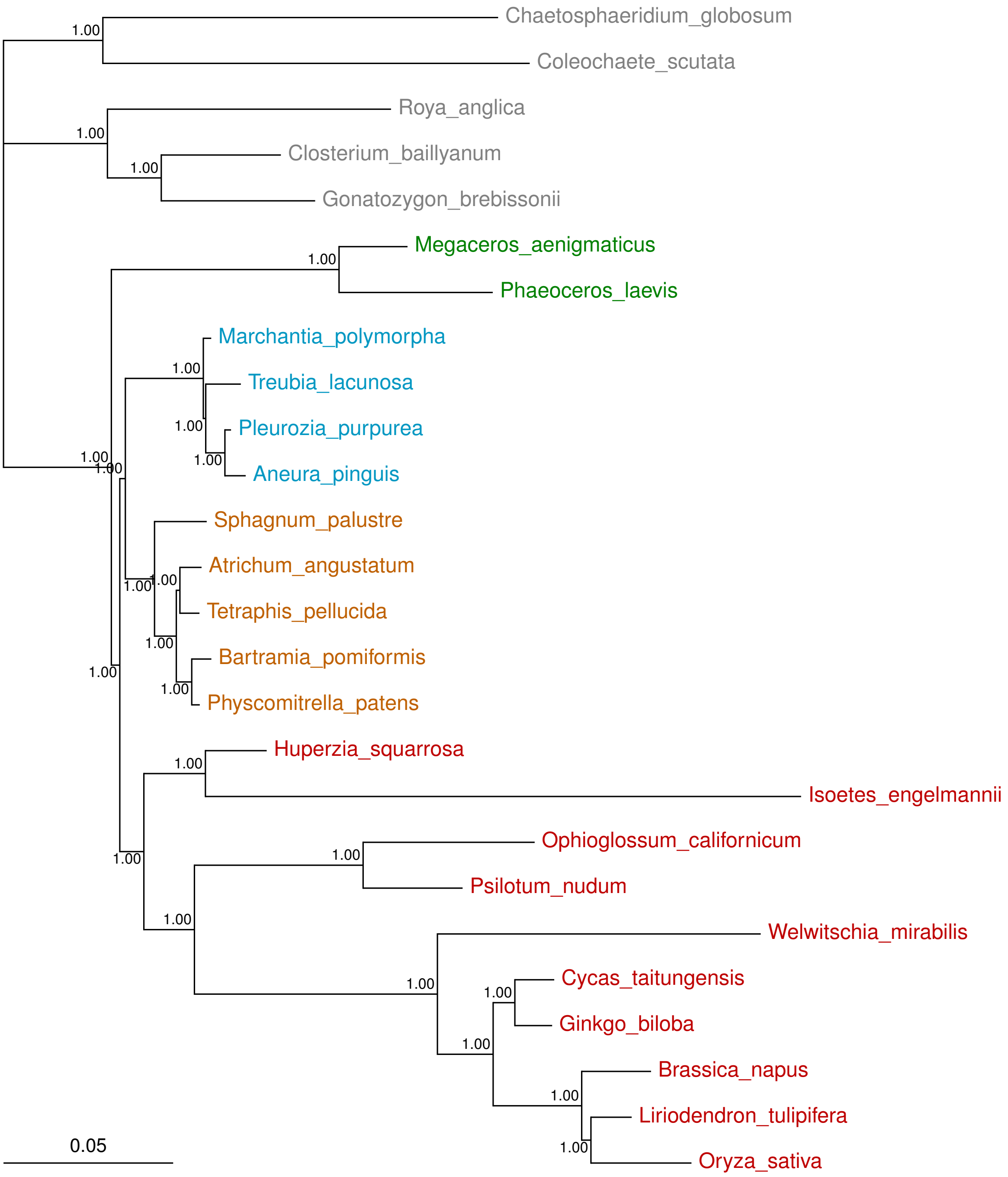

Figure S4 **36 genes/26 taxa** MCMC of the 36 gene degenerate nucleotide matrix using the model GTR+G and the NDCH2 model. The tree-heterogeneous composition model does not fit the expectation from posterior predictive simulations (p= 0.038). The consensus tree shows early-diverging hornworts (PP= 1.0). Runs r1. 3M generations, burnin= 21.000. $GTR + \Gamma_4 + F_{est}$ Marginal likelihood: $-\overline{L}_h$= 120030.89. Posterior predictive simulations of X^2: original statistic= 3690.7160, sample distribution= 2708.8691 to 4156.4237, p= 0.038

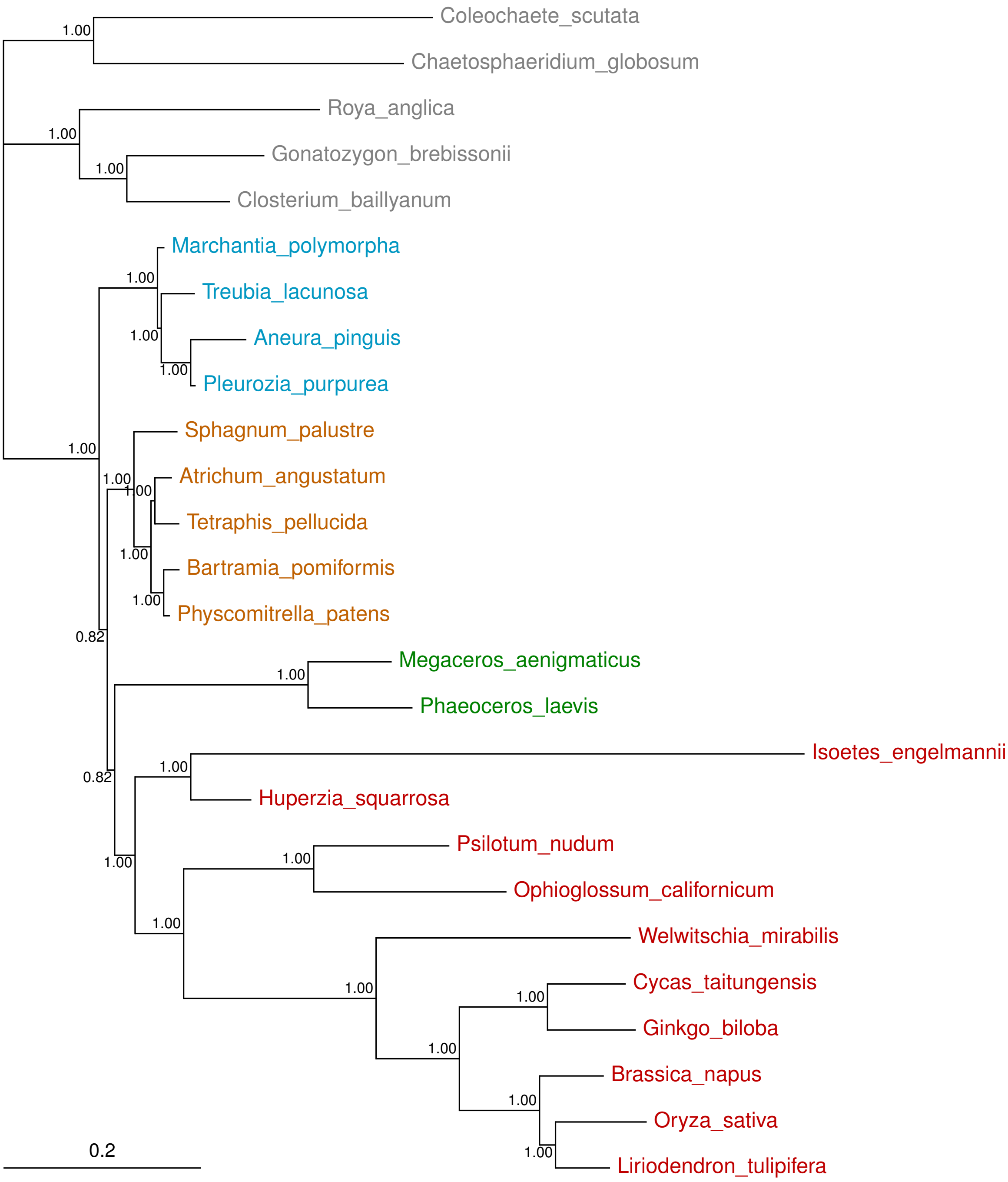

Figure S5 **36 genes/26 taxa** MCMC of the 36 gene amino acid matrix using the model stmtREV+G with one composition vector (CV1). The tree-homogeneous composition model does not fit the expectation from posterior predictive simulations (p= 0.0). The tree shows early-diverging liverworts (PP= 0.82). Runs x2. 1M generations, burnin= 6000. $stmtREV + \Gamma_4 + F_{est}$ Marginal likelihood: $-\overline{L}_h$= 146206.1180. ASDOSF= 0.0210. Posterior predictive simulations of X^2: original statistic= 3203.8039, sample distribution= 104.3447 to 216.2557, p= 0.0000

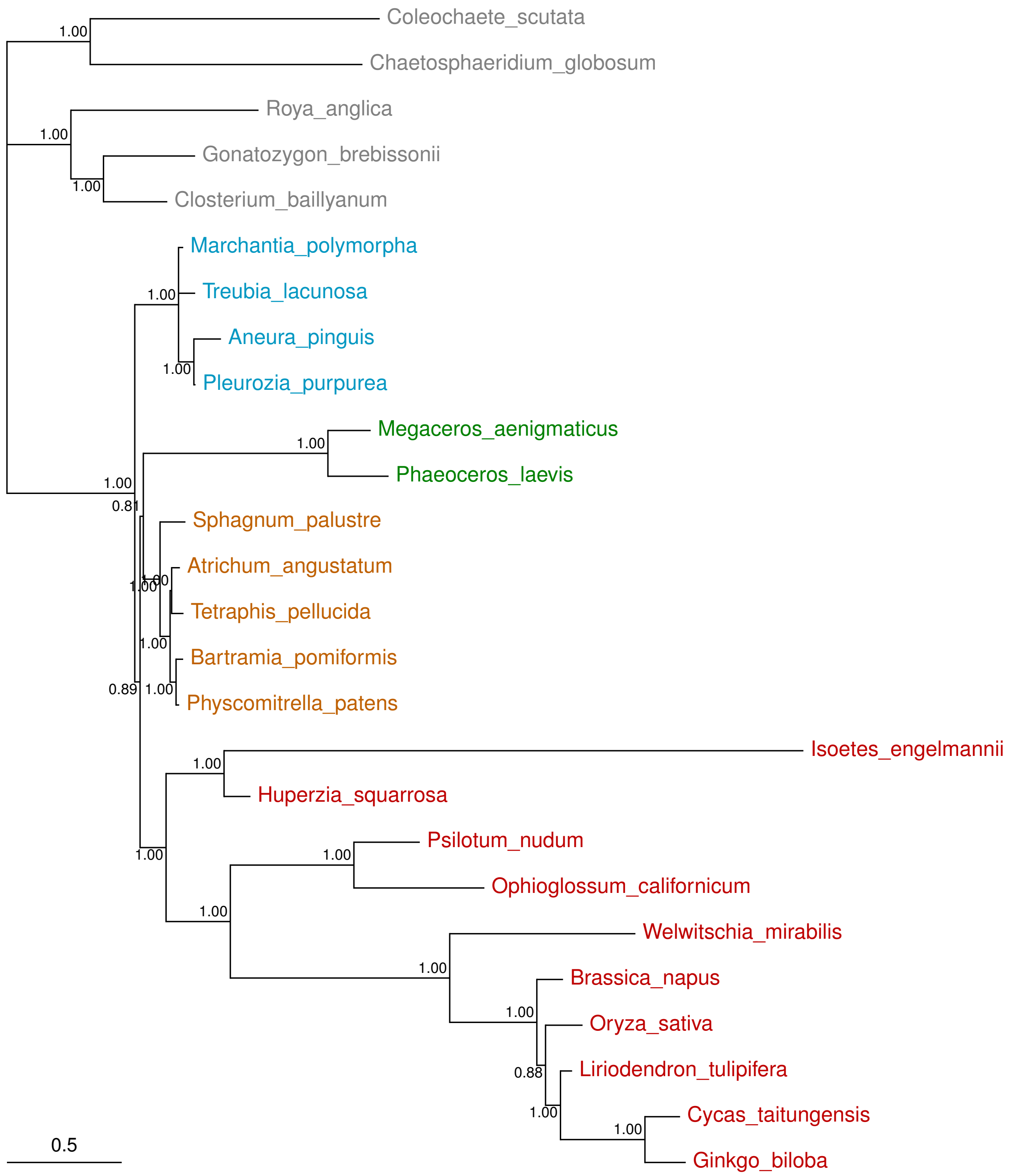

Figure S6 **36 genes/26 taxa** MCMC of the 36 gene amino acid matrix under the model CAT + stmtREV. The two runs converged on the same topology. N points: run1: 35846, run3: 36011; burnin: 31000; maxdiff= 0.146594). $CAT + stmtREV + \Gamma_4$ The tree shows early-diverging liverworts (PP= 0.89). The composition homogeneity (-comp) test had a p-value= 0.0 on both runs.

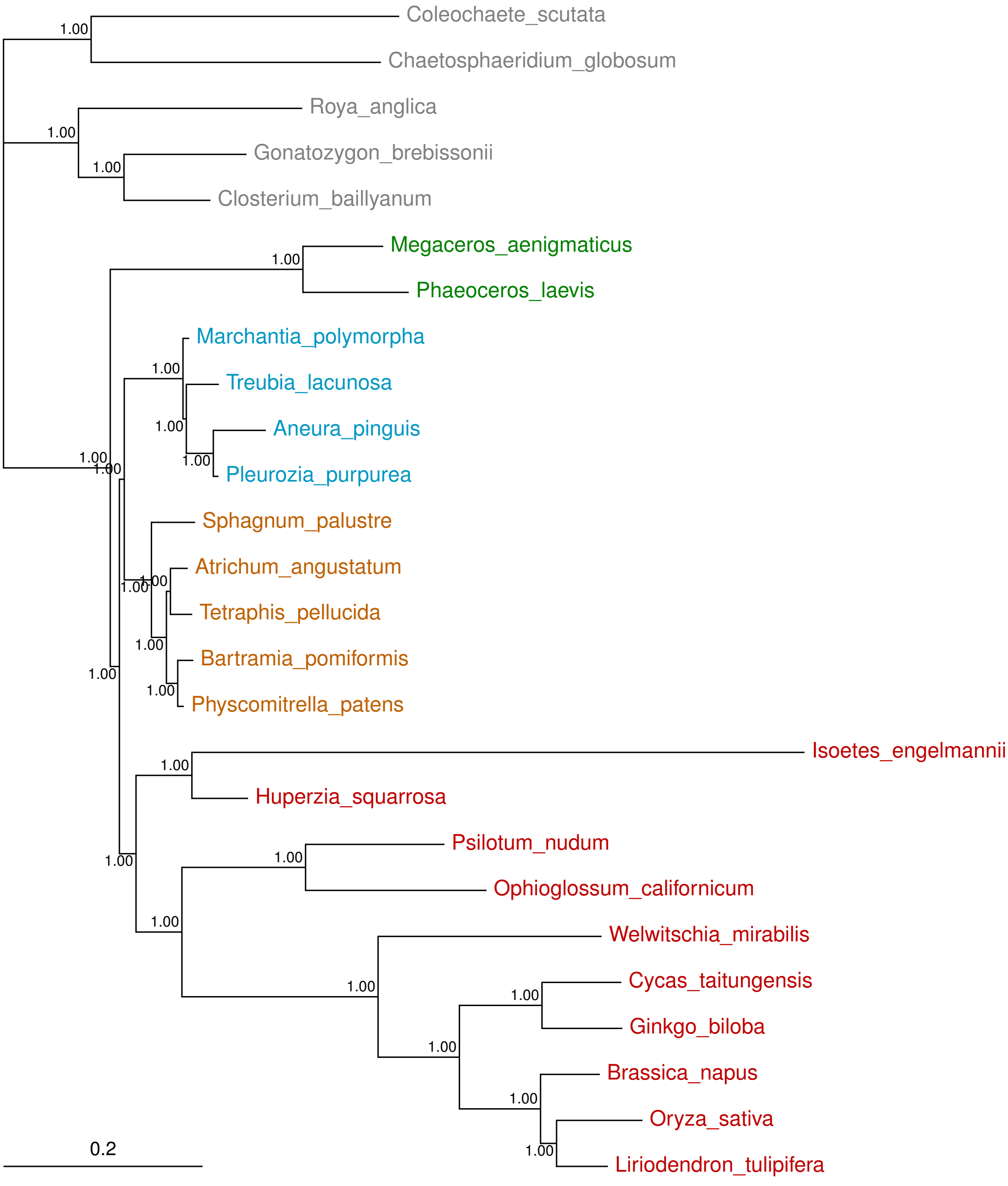

Figure S7 **36 genes/26 taxa** MCMC of the 36 gene amino acid matrix on p4 under the NDCH2 + stmtREV+G model. The tree-heterogeneous composition model fits the expectation from posterior predictive simulations (p= 0.1). The runs did not converge, the results from run 8 are reported. The tree obtained from run 8 shows early-diverging hornworts (PP= 1.0). Runs x1 (R8): 2M generations, burnin= 16000. $stmtREV + \Gamma_4 + F_{est}$ Marginal likelihood: $-\overline{L}_h$= 142829.112. Posterior predictive simulations of X^2: original statistic= 3203.8039, sample distribution= 2399.9329 to 3597.3115, p= 0.1022.
